# Digital Transformation and the Reconstruction of Nursing Professional Identity: Moving Beyond the Intangible Professional Project

**DOI:** 10.1111/jan.70482

**Published:** 2026-01-10

**Authors:** Gemma Stacey, Alice Sutton

**Affiliations:** ^1^ Nottingham Trent University Nottingham UK; ^2^ Parent of daughter with Wiedemann Steiner Syndrome London UK

## 1 Introduction

The healthcare sector is experiencing unprecedented digital transformation, fundamentally challenging established professional boundaries and traditional models of care delivery (Topol 2019). This paper argues that digital transformation presents nursing with a critical opportunity to establish a sustainable professional identity grounded in facilitative expertise, moving away from contested claims to exclusive knowledge domains toward recognition of nursing's distinctive capabilities in information navigation, relationship facilitation, collaborative expertise, and system navigation. Unlike medicine, which has successfully established clear jurisdictional boundaries around diagnosis and treatment, nursing's professional project remains contested and incomplete (Allen 2001).

The International Council of Nurses (ICN) defines nursing as “a profession dedicated to upholding everyone's right to enjoy the highest attainable standard of health, through a shared commitment to providing collaborative, culturally safe, people‐centred care and services” (ICN 2024). This definition emphasises that “nursing's practice is underpinned by a unique combination of science‐based disciplinary knowledge, technical capability, ethical standards, and therapeutic relationships.” However, digital transformation challenges how these therapeutic relationships are enacted and requires reconstruction of nursing practice within technologically mediated healthcare environments whilst maintaining the profession's commitment to “compassion, social justice and a better future for humanity.”

## 2 The Historical Context: Nursing's Contested Professional Project

Abbott's (1988) analysis of professional projects demonstrates how occupational groups establish legitimacy through control of specialised knowledge domains. Medicine exemplifies this model, having achieved professional dominance through clear diagnostic authority, prescriptive powers, and systematic control over medical knowledge (Freidson 2001). Nursing, however, presents a more complex case, struggling to define and defend unique professional territory within this same framework (Allen 2001).

This struggle manifests in several interrelated challenges. Nursing's holistic approach to care, whilst philosophically distinctive, has proven difficult to operationalise and measure in ways that clearly differentiate it from other healthcare roles (Allen 2001). The profession's emphasis on caring, whilst central to its identity, has been challenging to translate into exclusive professional claims given that caring is not unique to nursing (Davies 1995). Furthermore, nursing's historical subordination within medical hierarchies has complicated efforts to establish autonomous professional authority (Rafferty et al. 2007).

Contemporary scholarship has reconceptualized these challenges through the lens of professional identity formation. The International Society for Professional Identity in Nursing (ISPIN) characterises professional identity as “a sense of oneself, and in relationship with others, that is influenced by characteristics, norms, and values of the nursing discipline, resulting in an individual thinking, acting, and feeling like a nurse” (Godfrey and Young 2022). This definition shifts focus from territorial claims to identity construction, recognising professional identity development as an ongoing process influenced by socio‐historical contexts, educational preparation, workplace environments, generational trends, and prevailing culture.

Recent research demonstrates that professional identity encompasses multiple domains: values and ethics, knowledge, nurse leadership, and professional comportment. International studies reveal significant variations in how these domains manifest across countries, suggesting that nursing's professional identity challenge is not simply about establishing universal territorial claims, but about constructing coherent professional identity within diverse contexts (Mbalinda et al. 2024).

Yet nursing does possess distinctive characteristics that position it uniquely within healthcare systems. Through the nature of their interactions, nurses build trust with individuals, families and communities while gaining valuable insights into people's experiences of health and illness. This relational dimension represents a fundamental aspect of nursing's contribution to healthcare, even as it resists the territorial exclusivity that characterises traditional professional projects. The challenge, therefore, is not to abandon nursing's relational strengths in pursuit of territorial claims, but to reconstruct professional identity in ways that recognise these strengths as central to the profession's value.

## 3 Digital Disruption and Professional Identity Reconstruction

Contemporary healthcare digitalization intersects with nursing's professional identity challenges in ways that differ fundamentally from other healthcare professions. Recent systematic reviews reveal that technology growth, particularly electronic health records, compounds nursing's historical task‐centred focus, creating tension between efficiency demands and person‐centred care values (Forde et al. 2023).

For medicine, digital disruption threatens clearly established territories; AI diagnostic systems represent direct challenges to medical authority. For nursing, digital transformation may clarify professional identity by highlighting distinctive capabilities in navigation, interpretation, and relationship‐building within complex information environments.

Evidence from countries leading in healthcare digitalization demonstrates both opportunities and risks. Research reveals that when digital tools enhance rather than replace therapeutic relationships, nurses successfully adapt professional identity and maintain core professional values. Conversely, poorly designed digital systems undermine nursing's professional autonomy and force nurses into technical roles disconnected from core professional values (Kinnunen et al. 2023).

However, significant risks exist. Nursing's difficulty articulating unique value propositions means vulnerability to marginalisation within digital healthcare systems designed primarily around medical models. The reduction of nursing practice to algorithmic task allocation risks stripping away the therapeutic relationships that have historically defined the profession's contribution. The distinction between patient‐centred and person‐centred care becomes crucial, highlighting nursing's unique position within healthcare hierarchies that privilege functional outcomes over transformational wellness approaches (McCormack 2025).

## 4 The Incomplete Professional Project: Nursing's Identity Crisis

Nursing's professional project has been characterised by what Traynor (2013) describes as “persistent ambiguity” about the profession's core contribution. Whilst nursing has achieved significant advances in education, research capacity, and statutory recognition, it continues to struggle with fundamental questions about its unique domain.

This ambiguity manifests in several ongoing tensions: scope ambiguity with unclear boundaries between nursing and other healthcare roles; knowledge base contestation with ongoing debates about whether nursing represents a distinct discipline; value articulation difficulties in demonstrating economic value and outcome attribution; and hierarchical positioning with continued subordination within medical‐dominated systems despite formal professional autonomy. Nurses' intrinsic motivation and self‐identification can be barriers to fully embracing professional leadership roles. These pre‐existing identity challenges mean that digital transformation intersects with an already contested and incomplete professional project.

## 5 Digital Transformation as Professional Identity Opportunity: Four Core Domains

Digital transformation demands professional capabilities that align closely with nursing's fundamental strengths, offering pathways to establish clear professional identity. The following domains represent both theoretical constructs and practical competencies supported by international evidence.

### 5.1 Information Navigation

Information navigation emerges as a core nursing competency in digitally saturated healthcare environments but must transcend data interpretation to encompass genuine personal communication between persons. As patients gain access to unprecedented volumes of health data from wearable devices, genetic testing, AI‐generated risk assessments, and online health platforms, the challenge shifts from information scarcity to information interpretation.

Contemporary research demonstrates that nurses' informatics competencies are professional requirements essential for guaranteeing quality patient care and affect nurses' use of health information systems (Kinnunen et al. 2023). However, baseline assessments reveal that nursing students' perceived overall informatics competency remains at “somewhat competent” levels, with only 40.8% reporting competence, highlighting significant preparedness gaps. Canadian studies emphasise that entry‐to‐practice nursing informatics competencies must include use of digital health tools to support information synthesis in accordance with professional and regulatory standards (CASN 2025). Nurses are uniquely positioned to help patients and families navigate this complexity, not as gatekeepers controlling information access, but as skilled guides helping individuals understand data meaning in their specific contexts.

### 5.2 Relationship Facilitation

Relationship facilitation represents perhaps nursing's most distinctive contribution to digital healthcare. Cross‐national research reveals critical insights about maintaining therapeutic relationships across digital and physical care environments. Concept analysis demonstrates that therapeutic relational connection in telehealth refers to the intentional use of relationship connection between healthcare providers and patients as both parties work toward a therapeutic aim (Duffy et al. 2023). Research on group‐based telehealth interventions confirms that therapeutic relationships can be developed through factors requiring additional skills and effort compared with in‐person interactions.

Studies caution that digital relationship facilitation requires explicit skill development; assumptions that traditional interpersonal competencies automatically transfer to digital environments lead to reduced care quality. However, when implemented effectively, nurse‐led telehealth interventions successfully bridge healthcare gaps, with patients maintaining consistent relationships with designated nurses, leading to better communication and care coordination.

Contemporary concerns about reduced face‐to‐face interactions and the risk of creating functionally‐oriented rather than person‐centered practitioners highlight the importance of preserving nursing's irreplaceable capacity for creativity and emotional engagement that artificial intelligence fundamentally lacks (McCormack 2025). Digital transformation demands new forms of relational expertise: the ability to establish trust through video consultations, provide emotional support via digital platforms, and maintain continuity of caring relationships across hybrid care environments.

### 5.3 Collaborative Expertise

Collaborative expertise fundamentally reframes professional authority from hierarchical dominance toward horizontal partnership. This model acknowledges that in complex healthcare situations, optimal outcomes emerge from collaborative knowledge integration rather than individual expert decision‐making.

Recent scholarship supports this direction. Contemporary research on professional identity emphasises that all nurses should be and see themselves as leaders, exercising leadership to enhance the safety and efficacy of patient care while maintaining authentic leadership (Brewington et al. 2023). However, there remains a noticeable gap between this vision and reality, with international variations in how nurse leadership is conceptualised across healthcare systems.

Nurse practitioner‐led telehealth practices demonstrate that collaborative approaches can expand statewide access to care, extending services to high‐need areas and improving mental health access across diverse geographic regions (Fenton et al. 2024). Nurses practicing collaborative expertise work alongside patients as co‐experts in their own health, alongside other professionals as members of integrated teams, and alongside digital systems as partners in care delivery.

### 5.4 System Navigation

System navigation capitalises on nursing's traditional role as patient advocate whilst expanding it to encompass increasingly complex digital healthcare ecosystems. Modern healthcare requires individuals to navigate not only physical care environments but also digital platforms, insurance systems, community resources, and family support networks.

Contemporary research reveals that the rapidly expanding digitalization of healthcare has challenged nurses' competency requirements, necessitating increased educational programs for nurses on how to use digital devices and how to support patients to use digital services (Kinnunen et al. 2023). Scoping reviews of nurse practitioner‐led telehealth services confirm that these roles successfully impact access to care through appropriateness/ability to engage and availability/ability to reach services (Charalambous et al. 2024). Nurses practising system navigation help individuals understand how different components of the healthcare system work together, identify available resources, and coordinate care across multiple providers and platforms.

## 6 Preserving Personhood in Digital Environments

The challenge of maintaining authentic therapeutic relationships within digital systems requires explicit attention to preserving what cannot be reduced to algorithmic representations. This demands rebalancing technological competence with personal knowing, authentic vulnerability, and emotional engagement that characterises person‐centred practice. Nursing practice is fundamentally about providing professional care in the most personal health‐related aspects of people's lives and upholding dignity throughout life and at end of life, dimensions that cannot be fully captured by digital systems. Nursing education and practice development must address how to maintain recognition that persons possess agency for ethical and moral decision‐making through deep emotional engagement that transcends programmed responses.

## 7 Lived Experience: The Irreplaceable Human Dimension of Nursing Care

As someone who spends an inordinate amount of time in hospitals, I can testify to the fact that our recovery is not only based on the right medicines but also on the humanity which we experience on our road to getting better. This experiential perspective aligns with substantial empirical evidence. Recent qualitative research with clinical nurses reveals that quality care is fundamentally “holistic care” and “an interpersonal issue,” emphasising that therapeutic relationships form the essence of nursing practice (Tolosa‐Merlos et al. 2023). Patients report high satisfaction with nursing care quality, with satisfaction significantly associated with nurses' interpersonal skills, responsiveness, and person‐centered approaches.

Humans need more than just A + B = C, we need empathy. We need reassurance and encouragement. In our darkest hours, we look for a nurse to deliver what other professionals often cannot. Research across multiple healthcare settings confirms that patient experiences vary widely based on diverse elements affecting the quality of nursing care, with patients consistently emphasising the importance of nurses' communication, emotional support, and personalised attention.

That has been my experience on a children's ward; it has been my experience in a labour ward, and for my parents' generation of patients aged 80+, it has also been their experience in the community at home. These observations reflect broader patterns documented in recent patient experience research: studies reveal that patient experience scores in ambulatory settings reached five‐year highs in 2023, with patients reporting more positive perceptions when care emphasises empathy, personalization, and relationship‐building. Nurses are at the core of recovery and care; their compassion is essential and irreplaceable.

Patient and family perspectives provide crucial insights into nursing's distinctive contribution to healthcare that quantitative measures often fail to capture. The depth of nurse–patient relationships differs qualitatively from other healthcare professional interactions. These relationships are characterised not only by frequency of contact but by the nature of interactions that encompass humour, sympathy, and all the aspects of humanity that enable a holistic improvement in our health, reflecting the sustained engagement that characterises nursing practice.

This experiential knowledge underscores the risk that digitalisation poses if implemented without careful consideration of nursing's irreducible relational core. Importantly, families recognise both the potential and the limitations of digital transformation. There is acknowledgment that in a world of instant gratification and high public expectations for healthcare improvement, AI and digitalisation are drastically needed and a resource the health system must welcome, particularly for efficiency and reducing clinical errors. However, the same voices emphasise that recovery and care require more than technological efficiency; they require the compassion and humanity that nurses uniquely provide.

Contemporary research supports this dual perspective: systematic reviews examining digital health education and training confirm that while technological competencies are essential for modern nursing practice, they must be balanced with the preservation of core professional values and relational capabilities. Educational interventions that successfully develop digital competence do so by maintaining focus on nurses' therapeutic relationships with patients rather than purely technical skill acquisition.

This lived experience perspective validates theoretical concerns about maintaining authentic therapeutic relationships within increasingly digitised healthcare systems. It demonstrates that the evolution of digital capabilities within nursing must become a mutually beneficial and symbiotic relationship, where automation serves humanity rather than replacing it. The challenge for nursing's professional identity reconstruction lies in embracing technological advancement whilst preserving the essential human elements that patients and families consistently identify as irreplaceable.

## 8 Implications for Education and Practice Development

Professional identity reconstruction requires systematic changes that acknowledge nursing's unique position within the healthcare landscape whilst preparing practitioners for facilitative rather than authoritative professional roles. These changes must address both individual competency development and broader organisational culture transformation whilst explicitly resisting reductionist tendencies.

## 9 Identity Development

Identity development must become an explicit rather than implicit component of nursing education. International evidence demonstrates that professional identity formation requires transformative educational approaches including professional judgement, reasoning, critical self‐evaluation, and a sense of accountability (Mbalinda et al. 2024). Research confirms that participants believe learning ethics and values, nursing knowledge, and professional comportment are critical in developing professional identity (Owens and Denny 2024).

Educational transformation must address the philosophical foundations of person‐centred practice, helping students understand the importance of reconnecting with core professional values rather than allowing technological dominance to override relational foundations. This requires curriculum content that explicitly addresses nursing's professional history, including an honest examination of the profession's struggles with traditional professional project models and the opportunities that digital transformation presents for identity clarification.

## 10 Facilitation Skills

Facilitation skills represent a new category of professional competency requiring systematic development across multiple domains. Information navigation skills must include health informatics and data literacy alongside sophisticated communication capabilities for translating complex information into personally meaningful guidance whilst preserving authentic personhood.

Evidence demonstrates that structured competency frameworks combining health informatics literacy with communication capabilities improve nursing practice outcomes in digital environments. The 2025 Canadian Nursing Informatics Entry‐to‐Practice Competencies emphasise that nurses must be prepared to leverage digital health technologies while maintaining person‐centered care values (CASN 2025).

Digital relationship‐building requires understanding of virtual communication dynamics, online therapeutic presence, and technology‐mediated empathy, skills that differ significantly from traditional interpersonal competencies whilst maintaining capacity for authentic personal communication.

## 11 Leadership for Facilitation

Leadership for facilitation represents a fundamental departure from traditional nursing leadership models based on hierarchical authority toward approaches based on influence, collaboration, and systems thinking. International evidence reveals that nurse leaders who value their professional identity successfully facilitate change while maintaining authentic leadership focused on advancing all aspects of the profession (Brewington et al. 2023). Facilitative leaders must develop skills in consensus‐building, stakeholder engagement, and change management that emphasise participation rather than direction. This requires understanding organisational dynamics, policy development processes, and advocacy strategies that work through collaboration rather than confrontation whilst maintaining focus on preserving authentic caring relationships within technologically mediated systems.

## 12 Conclusion

Digital transformation intersects with nursing's historically contested professional identity in ways offering unprecedented opportunity for sustainable identity reconstruction. This paper argues that nursing's future lies not in defending exclusive knowledge claims never successfully established, but in embracing facilitative expertise aligned with collaborative digital healthcare models.

The ICN's (2024) definition of nursing provides a foundation for this reconstruction, emphasising that nursing practice fundamentally combines “science‐based disciplinary knowledge, technical capability, ethical standards, and therapeutic relationships.” As nurses integrate digital competencies while maintaining this unique combination, they construct a professional identity suited to contemporary healthcare realities.

International evidence demonstrates that successful digital transformation strengthens rather than threatens nursing professional identity when four conditions exist: (1) nurses possess clearly defined competencies in information navigation, relationship facilitation, collaborative expertise, and system navigation; (2) organisational systems support rather than undermine person‐centred care values during digitalization; (3) educational preparation explicitly addresses professional identity formation within digital contexts; and (4) leadership facilitates rather than directs technological change.

Patient and family voices remind us that while efficient digital processes and AI capabilities are needed and should be welcomed, at the core of all medicine is humanity, humans trying their best to help fellow humans. Nursing represents the practical application of this vocation, delivering not only medical care but the empathy, reassurance, encouragement, and compassion that patients consistently identify as essential and irreplaceable components of their recovery.

This represents not the failure of nursing's professional project, but its evolution toward a model more suited to both the profession's values and the realities of contemporary healthcare. The question is whether nursing can capitalise on this opportunity to establish a professional identity that maintains its caring essence whilst demonstrating irreplaceable value in technologically mediated healthcare environments. The answer may lie in listening carefully to those who experience nursing care most directly and allowing their insights to guide the profession's reconstruction of identity in the digital age.

## Funding

The authors have nothing to report.

## Conflicts of Interest

The authors declare no conflicts of interest.

## Data Availability

Data sharing not applicable to this article as no datasets were generated or analysed during the current study.
